# ﻿Name game conundrum: identical specific epithets in Microgastrinae (Hymenoptera, Braconidae)

**DOI:** 10.3897/zookeys.1183.111330

**Published:** 2023-11-07

**Authors:** Mostafa Ghafouri Moghaddam, Diana Carolina Arias-Penna, Minoo Heidari Latibari, Buntika A. Butcher

**Affiliations:** 1 Integrative Insect Ecology Research Unit, Department of Biology, Faculty of Science, Chulalongkorn University, Phaya Thai Road, Pathum Wan, Bangkok 10330, Thailand Chulalongkorn University Bangkok Thailand; 2 Bogotá, D.C., 111221, Colombia Unaffiliated Bogotá Colombia

**Keywords:** Binomial nomenclature, etymology, ICZN, scientific names, systematics

## Abstract

It is a privilege to recognize a new species and immortalize it with a name. Taxonomists may use etymologies recalling the sampling locality, habitat, species morphology, people (actor, writer, singer, politician, scientist), culture (customs, beliefs), fictional characters (gods, demons, cartoons), brands, ancient names, and others. Naming a species is a creative act that allows scientists to express their love for nature. By drawing on personal and cultural associations, species names are often imbued with far greater meaning than one might initially assume. Unconventional names for species can be an effective way to capture the imagination of the public and make the species memorable. In other words, species names can be both meaningful and whimsical. The central focus of this study was to pinpoint species in the subfamily Microgastrinae that share the same specific epithet that often creates confusion regarding which species is being referred to. The findings showed that 153 specific epithets were repeated representing 340 species in 52 genera, while the remaining 2,823 species have unique epithets. Three of the five categories proposed accommodate the majority of the etymologies: people (42%), morphology (27%), and geography (15%) whereas the categories of other (9%) and biology (7%) achieve the least representation. Approximately 95% of the same specific epithets had a single clear meaning, while for the remaining 5%, it was not possible to trace etymology. The study revealed that the average length of specific epithets was 9.01 letters, the longest contains 18 (*eliethcantillanoae*) while the shortest four (*eros* and *erro*). Additionally, most identical specific epithets were repeated two times (85.25% of the occurrences), although three (12.82%), five, six, and even nine (each one with 0.64%) repetitions were also found. Finally, a list of recommendations for taxonomists when faced with the task of naming a new species is provided.

## ﻿Introduction

The process of categorization is fundamental to the human experience, as it allows us to make sense of a chaotic, unpredictable environment, and to create order out of what may otherwise seem to be a random jumble of events (e.g., Mervis and Rosch 1981; Ashby and Maddox 2005; Pothos and Wills 2011). By classifying objects and phenomena, humans can elaborate predictions about how the world works, using this knowledge to make decisions and take action (McGarty et al. 2015). Humankind tends to group things into categories based on what they perceive to be the most convenient way of organizing them (i.e., categorization of animals, plants, programming languages, books, vehicles, clothing, food, musical instruments, etc.). This means that, even if the demarcations are not always logical, they are still deeply rooted in the way of looking at the world.

Linnaean nomenclature is the system of naming organisms developed by the Swedish scientist Carl Linnaeus, and the system uses a two-part Latin name for each species of living and fossil organism. The first part is termed the generic name and indicates the genus and the second one, the specific epithet, the species within the genus (Linnaeus 1751, 1758). Thus, the juxtaposition of these two names creates a unique combination that unambiguously identifies a species from any other similar species. This system has been in use for centuries (since 1751), and it is still the most widely accepted taxonomic nomenclature system by scientists today. Aside from the theoretical and practical advantages of unambiguous species names (Vink et al. 2012; Zeppelini et al. 2021), the Linnaean nomenclature provides the opportunity to use species names to honor the scientists who discovered them, the geographical locations they were found in, or even their interests. Scientific names also are chosen based on the morphological traits of the species, its environment, or the researcher’s creative ideas (Jozwiak et al. 2015; Ohl 2018). This is a way to make the work of classifying species more meaningful. As long as taxonomists adhere to a few basic grammatical rules regulating the derivation of etymologies, they can come up with a unique name for any species (Vendetti and Garland 2019). The creative potential of ascribing names is virtually boundless, presenting a wide range of possibilities and avenues for imaginative expression for the descriptor (Ohl 2018).

A small sampling of specific epithets used in parasitoid wasps is given below. Some epithets allude to species traits [e.g., *Wilkinsonelluscorpustriacolor* Arias-Penna, Zhang & Whitfield (Microgastrinae); it refers to the three colorations on the body; in Arias-Penna et al. 2014]; the locality of a species [e.g., *Ophiclypeuschiangmaiensis* Kang (Cardiochilinae); only known from Chiang Mai Province in Thailand; in Kang et al. 2023]; and biological aspects such as one of the levels of the tritrophic interactions, food plant-herbivore-parasitoid [e.g., *Cotesiatyphae* Fernández-Triana (Microgastrinae); *Typha* L. (Poales: Typhaceae) is the genus plant on which the herbivore lepidopteran host feeds on; in Kaiser et al. 2017]. More creatively, epithets may refer to renowned people [e.g., *Aleiodesgaga* Quicke & Butcher (Rogadinae); Stefani Joanne Angelina Germanotta (Lady Gaga) is an American singer, songwriter, and actress; in Butcher et al. 2012. *Conobregmabradpitti* Quicke & Butcher (Rogadinae); William Bradley (Brad) Pitt is an American actor and film producer; in Butcher et al. 2016. *Foenatopusnimaarkanii* Ghafouri Moghaddam & Rakhshani (Stephanidae); Nima Arkani-Hamed is an American-Canadian theoretical physicist of Iranian descent; in Ghafouri Moghaddam et al. 2019], a fictional cartoon character [e.g., *Masonapopeye* Quicke & Chaul (Masoninae); Popeye the Sailor Man cartoon; in Quicke et al. 2019], or simply represents a wordplay [e.g., *Hypomicrogasterzan* Valerio (Microgastrinae); the name is a random combination of letters without any meaning; in Valerio and Whitfield 2015].

The use of Latin and Latinized Greek words has been accepted and mandated by the International Code of Zoological Nomenclature (ICZN), which sets out the rules for naming organisms (ICZN 1999). This means that even though taxonomy is evolving and modernizing, Latin, an ancient language, is still the language of choice for naming organisms. The modern classification system has not changed significantly since its introduction centuries ago and is still valid today. Consequently, the Linnaean system is reliable with rules consistent over time.

Taxonomy is a field of science that fosters lively and sometimes contentious discussions. Notable among these is the emergence of novel methods for species classification, including photography-based (e.g., Ceríaco et al. 2016; Chaladze 2017; Faundez 2017), and COI sequence-based taxonomy (e.g., Meierotto et al. 2019; Sharkey et al. 2021; Chua et al. 2023). Furthermore, a plethora of heated debates have surfaced regarding the ICZN regulations (e.g., Dubois 2011; DuBay et al. 2020; Guedes et al. 2023; Pethiyagoda 2023; Roksandic et al. 2023; Slabin 2023; amongst many others), accompanied by a multitude of criticisms and recommendations voiced by researchers through social media platforms such as Taxacom, as well as in the form of published articles encompassing cases, declarations, opinions, comments, and correspondences.

Etymology refers to the study of the origin of words and their meanings (Jozwiak et al. 2015). This process involves tracking down the original description of the word by using dictionaries (Latin or Greek), employing the author’s prior knowledge of classical languages such as Greek and Latin, referring to previous works on the subject, or consulting webpages (e.g., Wikipedia, https://www.wikipedia.org/ and Wikispecies, https://species.wikimedia.org/) (Nilsson 2010).

The pivotal purpose of nomenclature is to establish a unique name for each taxon in the classification system. Maintaining consistency in the usage of organism names holds particular importance currently within digital environments. Binomial scientific names are unique identifiers within databases. In the context of online collaborative environments like the Global Biodiversity Information Facility (GBIF, https://www.gbif.org/), it becomes imperative to adhere to standardized criteria during the construction of these databases to ensure seamless utilization (see Welter-Schultes 2012). The ICZN does not impose any restrictions on using identical epithets for naming new species. Frequently, researchers should explore multiple sources to make sure they are looking at the correct species. At first glance, this task can be especially difficult and confusing when two species have identical epithets. To address this confusion, the ICZN (Articles 11, 23, 24, 32, 58, Recommendations 11A, 58A) encourages researchers to use similar names with slightly different spellings, or pairs of scientific and vernacular names to differentiate related species (ICZN 1999).

Etymologies are the stories behind words and their origins. By studying them, one can learn about the history of words and how their meanings have changed over time. This study aimed to delve into the choices made by authors for identical epithets when using etymologies for naming species, with a specific focus on Microgastrinae, a subfamily within the Braconidae that encompasses a total of 3,163 extant species distributed globally (Ghafouri Moghaddam et al. 2023).

## ﻿Materials and methods

Microgastrinae species names follow Fernández-Triana et al. (2020) which is the most comprehensive and up-to-date list of valid species for the subfamily. Taxonomic changes proposed in the publication were also adopted here. Additionally, all newly discovered species until August 2023 were included. The number of currently known microgastrine species was retrieved from the website Microgastrinae Wasps of the World (Ghafouri Moghaddam et al. 2023). Detailed information from the original description of Microgastrinae species can be found in Fernández-Triana et al. (2020).

This research is laser-focused on a specific subset—species that share identical specific epithets in their names. This subset represents part of the whole diversity of microgastrine names, making the data manageable and easier to compare and analyze. It is worth pointing out that there are instances of partial matches observed in specific epithets. This is attributed to their variations in the spelling, and using the surname or full name, which subsequently these examples were excluded from the analysis (e.g., *andybenneti*; *brasiliensis* and *braziliensis*; *brevicarinata* and *brevicarinis*; *gavinbroadi*; *cameronae*, *sydneyae*, and *sydneycameronae*; and *mikesharkeyi*).

Species etymologies were classified into five broad categories (in alphabetical order): those referring to (1) biology (host associations, habitat, or some behavioral adaptation), (2) geography (distribution of the species), (3) morphology (morphological traits including size, color, or shape), (4) other (epithets that are puns, arbitrary combinations of letters, names that refer to a legend, names of gods or deities, and many others), and (5) people (fictional characters, scientists, actors, singers, political officials, beloved ones, other people).

The category to which each of the epithets belongs was determined by consulting the original descriptions under the section Etymology. In cases where this section was missing, the text was scrutinized for any clues that provided insight into the meaning of the species epithet.

## ﻿Results

**Figure 1.**
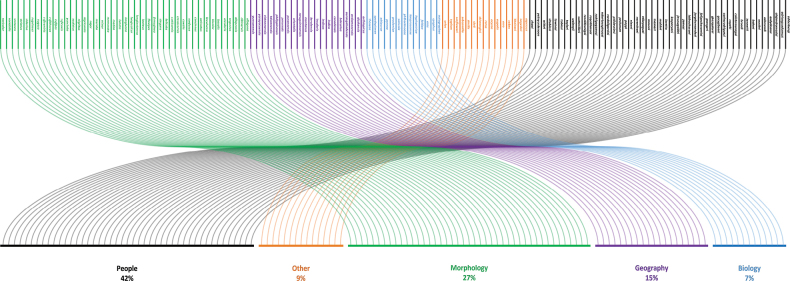
The five categories used for the classification of the identical specific epithets in the subfamily Microgastrinae (Hymenoptera, Braconidae) display their respective frequency of use. Colored bars represent categories, blue = biology, purple = geography, green = morphology, orange = others, and black = people.

In total, 153 identical specific epithets were found in Microgastrinae which represent 340 species in 52 genera. The remaining 2,823 species displayed unique epithets. The level of repetition of epithets was low (10.7%) which could potentially lead to inaccurate results when analyzing the specific epithet distributions within the subfamily. The categories of people (42%, corresponding to 142 spp.), morphology (27%, 92 spp.), and geography (15%, 50 spp.) are the ones with the most repeated specific epithets whereas other (9%, 30 spp.) and biology (7%, 26 spp.) obtained the least number of repetitions (Fig. 1). The vast majority (95%) of repeated specific epithets have an unambiguous meaning and only a small fraction (5%) have an uncertain definition and probably multiple meanings.

**Figure 2.**
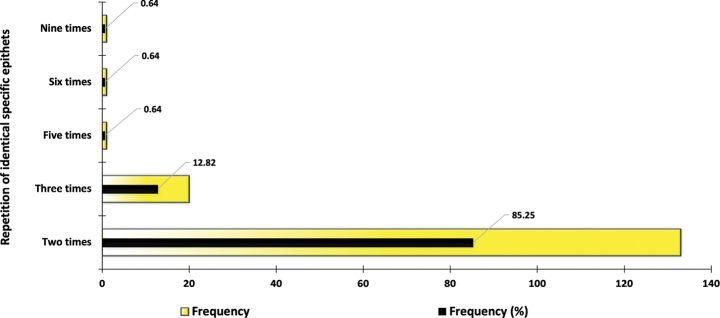
Frequency and percentage frequency distribution of identical and repeated specific epithets within the subfamily Microgastrinae (Hymenoptera, Braconidae).

The vast majority of specific epithets were repeated two times (85.25% of the occurrences) followed by three times (12.82% of the occurrences). In contrast, a small number of specific epithets were repeated five, six, and even nine times but each one accounted for less than 1% of the occurrences (Fig. 2). The specific epithets with the highest number of repetitions were *masoni* (in nine genera), *orientalis* (in six genera), and *nixoni* (in five genera).

**Figure 3.**
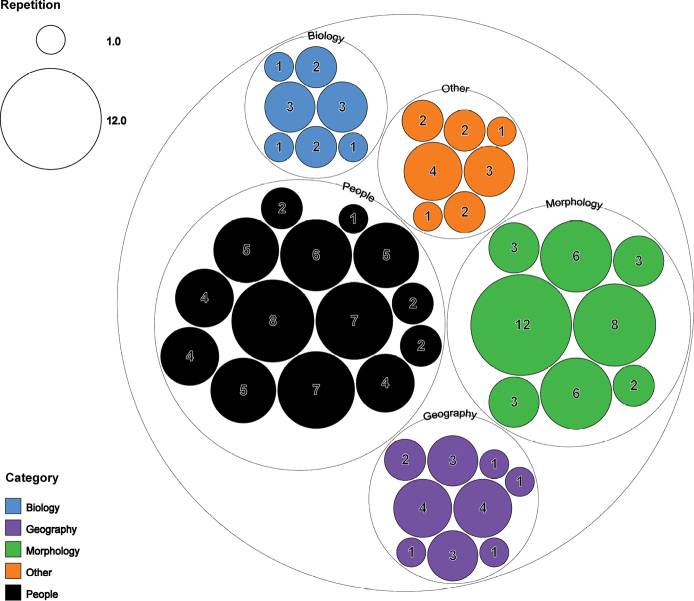
Occurrences of identical specific epithets with the same number of alphabetic characters across the five categories within the subfamily Microgastrinae (Hymenoptera, Braconidae). In each circle, the number indicates the number of letters that compose the specific epithet and the relative size of the circle represents the number of species in each group.

As to the length of the specific epithet (number of alphabetic characters), the average length was nine. The longest epithet, *eliethcantillanoae*, contained 18 alphabetic characters and belonged to the people category. The shortest epithets, *eros* (in the biology category) and *erro* (in the other category), each contained only four letters. By analyzing each category, the highest number of specific epithets repeated with the same number of letters was located in morphology (eight letters in 12 species) followed by the people category (seven letters in eight species), and the geography category (eleven letters in four species) (Fig. 3).

When analyzed by category, the same pattern was observed, a large number of specific epithets was repeated twice, meaning assigned to only two genera (Table 1). Thus, the highest percentage of occurrences was in the biology and the other categories, where all of the specific epithets (100%) were repeated twice. In contrast, the category of people had the lowest percentage (15%). Identical specific epithets were also repeated three times but to a lesser extent and only in three categories, geography (25%), morphology (14%), and people (13%). People is the only category with specific epithets replicated two, three, five, and nine times.

**Table 1.** The five categories into which identical specific epithets were classified within the subfamily Microgastrinae (Hymenoptera, Braconidae) with their respective frequency percentages.

Category	Two times	Three times	Five times	Six times	Nine times
**Biology**	100%	–	–	–	–
**Geography**	70%	25%	–	5%	–
**Morphology**	86%	14%	–	–	–
**Other**	100%	–	–	–	–
**People**	82%	13%	2%	–	2%

The identical specific epithets in the Microgastrinae species are listed alphabetically as follows.

### ﻿List of identical specific epithets within the subfamily Microgastrinae

***achterbergi***

**Etymology.** The epithet honors the Dutch braconidologist, Cornelis (Kees) van Achterberg, whose work contributes to the knowledge of Braconidae of the world, as well as other Hymenoptera groups. He is a world-renowned hymenopterologist and his work is exemplary and truly remarkable. The suffix -*i* added to the term is a masculine Latin genitive.

**Taxa.** The epithet has been used in three genera: *Bulukaachterbergi* Austin, 1989; *Choerasachterbergi* Narendran, 1998; and *Forniciaachterbergi* Yang & Chen, 2006.

**Category.** People.

***adrianguadamuzi***

**Etymology.** The epithet refers to Adrian Guadamuz for his contributions to the Área de Conservación Guanacaste (ACG) both in the Programa de Parataxónomos and the plant inventory of ACG in Costa Rica (Central America). The suffix -*i* added to the term is a masculine Latin genitive.

**Taxa.** The epithet has been used in three genera: *Alphomelonadrianguadamuzi* Fernández-Triana & Shimbori, 2023; *Apantelesadrianguadamuzi* Fernández-Triana, 2014; and *Microplitisadrianguadamuzi* Fernández-Triana & Whitfield, 2015.

**Category.** People.

***aethiopicus***

**Etymology.** The word *aethiopicus* contains two parts, *Aethiopia* the Latin spelling of Ethiopia, an east African country located in the Somali peninsula, and the Latin suffix -*icus* which means belonging to. In taxonomy, the term *aethiopicus* describes a species that is native to or has characteristics associated with Ethiopia.

**Taxa.** The epithet has been used in two genera: *Jenopappiusaethiopicus* (de Saeger, 1944); and *Parapantelesaethiopicus* (Wilkinson, 1931).

**Category.** Geography.

***alaskensis***

**Etymology.** The term *alaskensis* contains two parts, Alaska, one of the states in the United States, and the Latin suffix -*ensis* which means belonging to or inhabiting. In taxonomy, the term *alaskensis* literally means belonging to Alaska or inhabiting Alaska.

**Taxa.** The epithet has been used in two genera: *Microplitisalaskensis* Ashmead, 1902; and *Protapantelesalaskensis* Ashmead, 1902.

**Category.** Geography.

***albigena***

**Etymology.** The term *albigena* comes from two Latin words, *albus* meaning white and *gena* meaning cheek. In taxonomy, *albigena* describes a wasp with white cheeks.

**Taxa.** The epithet has been used in two genera: *Glyptapantelesalbigena* Fagan-Jeffries, Bird & Austin, 2022; and *Protapantelesalbigena* Abdoli, Fernández-Triana & Talebi, 2021.

**Category.** Morphology.

***albinervis***

**Etymology.** The term *albinervis* is made up of two Latin words, *albi* meaning white and *nervis* meaning veins. In taxonomy, *albinervis* indicates the white veins of the species.

**Taxa.** The epithet has been used in two genera: *Apantelesalbinervis* (Cameron, 1904); and *Iconellaalbinervis* (Tobias, 1964).

**Category.** Morphology.

***albipennis***

**Etymology.** The term *albipennis* comes from two Latin words, *albi m*eaning white and *pennis* which means feather or wing. In taxonomy, the term *albipennis* describes a species with white wings.

**Taxa.** The epithet has been used in two genera: *Dolichogenideaalbipennis* (Nees, 1834); and *Microplitisalbipennis* Abdinbekova, 1969.

**Category.** Morphology.

***alejandromasisi***

**Etymology.** The name refers to Alejandro Masis for his relentless efforts to administrate and protect the Área de Conservación Guanacaste (ACG) in Costa Rica (Central America). The suffix -*i* added to the term is a masculine Latin genitive.

**Taxa.** The epithet has been used in two genera: *Apantelesalejandromasisi* Fernández-Triana, 2014; and *Dolichogenideaalejandromasisi* Fernández-Triana & Boudreault, 2019.

**Category.** People.

***andamanensis***

**Etymology.** The term *andamanensis* contains two parts, Andaman, the Andaman Islands, an archipelago in the Bay of Bengal between India and Myanmar, and the Latin suffix -*ensis* which means belonging to or of the place of. In taxonomy, *andamanensis* indicates that the species is native to the Andaman Islands.

**Taxa.** The epithet has been used in two genera: *Diolcogasterandamanensis* Gupta & Fernández-Triana, 2015; and *Forniciaandamanensis* Sharma, 1984.

**Category.** Geography.

***andydeansi***

**Etymology.** The name honors Andrew (Andy) Robert Deans, an American entomologist, who has made exceptional contributions to the knowledge of Evaniidae (Hymenoptera) on a global scale. The suffix -*i* added to the term is a masculine Latin genitive.

**Taxa.** The epithet has been used in three genera: *Alphomelonandydeansi* Fernández-Triana & Shimbori, 2023; *Glyptapantelesandydeansi* Arias-Penna, 2019; and *Iconellaandydeansi* Fernández-Triana, 2013.

**Category.** People.

***angustus***

**Etymology.** The term *angustus* derives from the Latin verb *ango* which means to narrow, to constrict, or to tighten. In taxonomy, *angustus* refers to the narrow length of a particular morphological trait. Thus, the narrow areolation on the propodeum (in *Apanteles* Foerster) or a very narrow mesosoma and metasoma (in *Choeras* Mason).

**Taxa.** The epithet has been used in two genera: *Apantelesangustus* Liu & Chen, 2020; and *Choerasangustus* Song & Chen, 2014.

**Category.** Morphology.

***areolaris***

**Etymology.** The term *areolaris* comes from the Latin word *areola* which means small open space or little courtyard. In taxonomy, *areolaris* refers to a species that has a body part covered with areolate, a type of surface sculpturing.

**Taxa.** The epithet has been used in two genera: *Hypomicrogasterareolaris* (Blanchard, 1947); and *Microgasterareolaris* Thomson, 1895.

**Category.** Morphology.

***ashmeadi***

**Etymology.** The name is a tribute to the American entomologist William Harris Ashmead (1855–1908), one of the most important hymenopterologist authorities from the 19^th^ century. The suffix -*i* added to the term is a masculine Latin genitive.

**Taxa.** The epithet has been used in two genera: *Diolcogasterashmeadi* Saeed, Austin & Dangerfield, 1999; and *Glyptapantelesashmeadi* (Wilkinson, 1928).

**Category.** People.

***aspersus***

**Etymology.** The term *aspersus* is a Latin adjective that means sprinkled or sprayed with. In taxonomy, *aspersus* reflects the idea of something being sprinkled or scattered over a surface. In *Apanteles*, *aspersus* refers to the spines on the outer side of the third tibia as rather sparse while in *Glyptapenteles* Ashmead, refers to the widely dispersed distribution of the species.

**Taxa.** The epithet has been used in two genera: *Apantelesaspersus* Liu & Chen, 2020; and *Glyptapantelesaspersus* Fagan-Jeffries, Bird & Austin, 2022.

**Category.** Other.

***aurangabadensis***

**Etymology.** The term *aurangabadensis* contains two parts, Aurangabad, an Indian city belonging to the Maharashtra state, and the Latin suffix -*ensis* which means belonging to or of the place of. In taxonomy, *aurangabadensis* indicates that the species was first discovered or described in the area around Aurangabad.

**Taxa.** The epithet has been used in two genera: *Apantelesaurangabadensis* Rao & Chalikwar, 1970; and *Diolcogasteraurangabadensis* Fernández-Triana, 2019.

**Category.** Geography.

***austini***

**Etymology.** The name honors Andrew (Andy) Donald Austin, an Australian hymenopterologist for his outstanding contributions to the knowledge of Microgastrinae of Australia. The suffix -*i* added to the term is a masculine Latin genitive.

**Taxa.** The epithet has been used in two genera: *Glyptapantelesaustini* Fagan-Jeffries & Bird, 2022; and *Miropotesaustini* Fernández-Triana & Whitfield, 2014.

**Category.** People.

***autographae***

**Etymology.** The epithet *autographae* refers to the looper moth genus, *Autographa* Hübner (Noctuidae: Plusiinae), which is the herbivore host used by these microgastrine wasps.

**Taxa.** The epithet has been used in two genera: *Cotesiaautographae* (Muesebeck, 1921); and *Microplitisautographae* Muesebeck, 1922.

**Category.** Biology.

***bageshri***

**Etymology.** The term *bageshri* is likely a reference to the Indian classical music raga called Bageshri. The exact reason for this name choice is unclear, but it may be a nod to the Indian origins of the wasp species or to the sweet and soothing nature of the raga, which may reflect the behavior or appearance of the wasp.

**Taxa.** The epithet has been used in two genera: *Dolichogenideabageshri* Sathe, Inamdar & Dawale, 2003; and *Microplitisbageshri* Sathe, Inamdar & Dawale, 2003.

**Category.** Other.

**Note.** Both species are under the status of unavailable names (see more detail in Fernández-Triana et al. 2020).

***bakeri***

**Etymology.** The name is dedicated to Charles Fuller Baker (1872–1927), an American entomologist, botanist, and agronomist who contributed greatly to natural science knowledge in the late 19^th^ and early 20^th^ centuries. The suffix -*i* added to the term is a masculine Latin genitive.

**Taxa.** The epithet has been used in two genera: *Diolcogasterbakeri* (Muesebeck, 1922); and *Dolichogenideabakeri* (Wilkinson, 1932).

**Category.** People.

***balearica***

**Etymology.** The word *balearica* refers to the Balearic Islands, a group of islands located in the western Mediterranean Sea.

**Taxa.** The epithet has been used in two genera: *Cotesiabalearica* Shaw & Colom, 2022; and *Microgasterbalearica* Marshall, 1898.

**Category.** Geography.

***basalis***

**Etymology.** The word *basalis* derives from two Latin words, *basis* meaning foundation or base, and the suffix -*alis* which indicates related to or pertaining to. In taxonomy, *basalis* describes a species with a basal or fundamental form, structure, or behavior. In *Microplitis*, probably refers to the medial longitudinal groove in the basal of the first tergite while in *Snellenius* refers to white metasoma in lateral views toward the base.

**Taxa.** The epithet has been used in two genera: *Microplitisbasalis* (Bingham, 1906); and *Snelleniusbasalis* (Walker, 1874).

**Category.** Morphology.

***bennetti***

**Etymology.** The name refers to Andrew (Andy) Michael Reeve Bennett, a Canadian entomologist and one of the world’s authorities on ichneumonid wasps. The suffix -*i* added to the term is a masculine Latin genitive.

**Taxa.** The epithet has been used in two genera: *Deuterixysbennetti* Whitfield, 1985; and *Sendaphnebennetti* Fernández-Triana & Whitfield, 2014.

**Category.** People.

***bhairavi***

**Etymology**. The term *bhairavi* comes from Bhairavi, a Hindu goddess whose name means terror or awe-inspiring. The term Bhairavi can also refer to the Hindustani classical music from the northern regions of India. It is a type of raga, a melodic framework for improvisation in Indian classical music. The exact reason for this name choice is unclear, but it may be a nod to the Indian origins of the wasp species (e.g., *Cotesiabhairavi* (Sathe & Inamdar, 1991)) that was collected in Sikkim, a state in northeastern India although the species is deposited in the collection of Shivaji University, Kolhapur (India). Another interpretation is that the term refers to the intense and powerful nature of the raga, which may reflect the behavior or appearance of the wasp.

**Taxa.** The epithet has been used in two genera: *Cotesiabhairavi* (Sathe & Inamdar, 1991); and *Parenionbhairavi* Sathe, Inamdar & Dawale, 2003.

**Category.** Other.

**Note.***Parenionbhairavi* Sathe, Inamdar & Dawale, 2003 is under the status of unavailable names (see more detail in Fernández-Triana et al. 2020).

***bicolor***

**Etymology.** The term *bicolor* contains two Latin words, the prefix -*bi* which means two and the noun *color* which is a cognate of the English word. In taxonomy, *bicolor* is often used as an adjective to describe a species that has two distinct colors or color patterns on the body or specific body parts.

**Taxa.** The epithet has been used in three genera: *Dolichogenideabicolor* Song & Chen, 2004; *Pholetesorbicolor* (Nees, 1834); and *Snelleniusbicolor* Shenefelt, 1968.

**Category.** Morphology.

***brevivena***

**Etymology.** The term *brevivena* contains two Latin words, *brevi* which means short and *vena* which means vein. In taxonomy, *brevivena* refers to a species with short veins.

**Taxa.** The epithet has been used in two genera: *Apantelesbrevivena* Liu & Chen, 2015; and *Diolcogasterbrevivena* Zeng & Chen, 2011.

**Category.** Morphology.

***broadi***

**Etymology.** The name honors the British Gavin R. Broad, one of the world’s leading authorities of our time on the study of Ichneumonidae. The suffix -*i* added to the term is a masculine Latin genitive.

**Taxa.** The epithet has been used in two genera: *Dolichogenideabroadi* Rousse, 2013; and *Sendaphnebroadi* Fernández-Triana & Whitfield, 2014.

**Category.** People.

***calixtomoragai***

**Etymology.** The epithet refers to Calixto Moraga for his contributions to the Área de Conservación Guanacaste (ACG) in the Programa de Parataxónomos in Costa Rica (Central America). The suffix -*i* added to the term is a masculine Latin genitive.

**Taxa**. The epithet has been used in two genera: *Alphomeloncalixtomoragai* Fernández-Triana & Shimbori, 2023; and *Apantelescalixtomoragai* Fernández-Triana, 2014.

**Category.** People.

***canadensis***

**Etymology.** The term *canadensis* consists of two words, Canada, a country in North America, and the Latin suffix -*ensis* which means belonging to or inhabiting. In taxonomy, *canadensis* refers to a species that is native to or found in Canada.

**Taxa**. The epithet has been used in three genera: *Clarkinellacanadensis* Mason, 1981; *Iconellacanadensis* Fernández-Triana, 2013; and *Microgastercanadensis* Muesebeck, 1922.

**Category.** Geography.

***capeki***

**Etymology.** The name honors Miroslav Čapek (1927–2008), a Czech braconidologist from the 20^th^ century in appreciation of his relevant entomological contributions. The suffix -*i* added to the term is a masculine Latin genitive.

**Taxa.** The epithet has been used in two genera: *Glyptapantelescapeki* (Györfi, 1955); and *Microplitiscapeki* Nixon, 1970.

**Category.** People.

***carinatus***

**Etymology.** The term *carinatus* derives from the Latin word *carina* which means keel or ridge. In taxonomy, *carinatus* describes a species that has a distinct ridge or keel on a specific body part such as mesosoma or metasoma.

**Taxa.** The epithet has been used in two genera: *Glyptapantelescarinatus* (Szépligeti, 1913); and *Microplitiscarinatus* Song & Chen, 2008.

**Category.** Morphology.

***carlosrodriguezi***

**Etymology.** The name refers to Carlos Rodríguez in recognition of his tireless efforts with the Programa de Ecoturismo in the Área de Conservación Guanacaste (ACG) in Costa Rica (Central America). The suffix -*i* added to the term is a masculine Latin genitive.

**Taxa.** The epithet has been used in two genera: *Apantelescarlosrodriguezi* Fernández-Triana, 2014; and *Pseudapantelescarlosrodriguezi* Fernández-Triana & Whitfield, 2014.

**Category.** People.

***carolinacanoae***

**Etymology.** The epithet refers to Carolina Cano for her contributions to the Área de Conservación Guanacaste (ACG) in the Programa de Parataxónomos in Costa Rica (Central America). The suffix -*ae* added to the term is a feminine Latin genitive.

**Taxa**. The epithet has been used in two genera: *Alphomeloncarolinacanoae* Fernández-Triana & Shimbori, 2023; and *Apantelescarolinacanoae* Fernández-Triana, 2014.

**Category.** People.

***cebes***

**Etymology.** The epithet is probably dedicated to Cebes, an Ancient Greek philosopher from Thebes who was a disciple of Socrates.

**Taxa.** The epithet has been used in two genera: *Apantelescebes* Nixon, 1965; and *Microplitiscebes* Nixon, 1970.

**Category.** Other.

***christerhanssoni***

**Etymology.** The epithet honors the Swedish chalcidologist, Christer Hansson, whose work contributes to the knowledge of Eulophidae of the world. The suffix -*i* added to the term is a masculine Latin genitive.

**Taxa**. The epithet has been used in two genera: *Alphomelonchristerhanssoni* Fernández-Triana & Shimbori, 2023; and *Glyptapanteleschristerhanssoni* Arias-Penna, 2019.

**Category.** People.

***concinnus***

**Etymology.** The term *concinnus* derives from the Latin verb *concinnare* which means to make ready or to put in order. In taxonomy, *concinnus* describes a species that has a well-ordered or harmonious appearance, often about a morphological trait or coloration.

**Taxa.** The epithet has been used in two genera: *Apantelesconcinnus* Statz, 1938; and *Glyptapantelesconcinnus* (Muesebeck, 1958).

**Category.** Morphology.

**Note.***Apantelesconcinnus* Statz, 1938 is a species only known from fossils (see more detail in Fernández-Triana et al. 2020).

***confusus***

**Etymology.** The Latin word *confusus* means confused or mixed up. In taxonomy, *confusus* likely refers that initially, the species was unclear or confusing (referring to more or less merged second + third tergites), as is the case with many newly discovered Microgastrinae species.

**Taxa.** The epithet has been used in two genera: *Microplitisconfusus* Muesebeck, 1922; and *Pholetesorconfusus* Liu & Chen, 2016.

**Category.** Morphology.

***coxalis***

**Etymology.** The term *coxalis* derives from two Latin words, *coxa* which means hip and the suffix -*alis* which means related to or pertaining to. In taxonomy, *coxalis* refers to a species whose coxa has a particularity either in size, color, or sculpturing.

**Taxa.** The epithet has been used in two genera: *Apantelescoxalis* Szépligeti, 1911; and *Diolcogastercoxalis* (de Saeger, 1944).

**Category.** Morphology.

***crassicornis***

**Etymology.** The word *crassicornis* is made up of two Latin words, *crassus* which means thick or fat and *cornis* which means horn. In taxonomy, *crassicornis* describes a species whose antennae are thick or club-shaped that resemble horns.

**Taxa.** The epithet has been used in two genera: *Apantelescrassicornis* (Provancher, 1886); and *Microgastercrassicornis* Ruthe, 1860.

**Category.** Morphology.

***curticornis***

**Etymology.** The term *curticornis* is composed of two Latin words, *curtus* which means short or truncated and *cornis* which means horn. In taxonomy, *curticornis* describes a species with short or truncated antennae that resemble horns.

**Taxa.** The epithet has been used in two genera: *Diolcogastercurticornis* (Granger, 1949); and *Venanidescurticornis* (Granger, 1949).

**Category.** Morphology.

***delicata***

**Etymology.** The Latin word *delicata* means pleasing, delightful, dainty, or fine. In taxonomy, *delicata* refers to the general appearance of the body which is delicate and slender.

**Taxa.** The epithet has been used in two genera: *Austrocotesiadelicata* Austin & Dangerfield, 1992; and *Cotesiadelicata* (Howard, 1897).

**Category.** Morphology.

***diniamartinezae***

**Etymology.** The epithet refers to Dinia Martínez for her contributions to the Área de Conservación Guanacaste (ACG) in the Programa de Parataxónomos in Costa Rica (Central America). The suffix -*ae* added to the term is a feminine Latin genitive.

**Taxa**. The epithet has been used in two genera: *Alphomelondiniamartinezae* Fernández-Triana & Shimbori, 2023; and *Apantelesdiniamartinezae* Fernández-Triana, 2014.

**Category.** People.

***duvalierbricenoi***

**Etymology.** The epithet refers to Duvalier Briceño for his contributions to the Área de Conservación Guanacaste (ACG) in the Programa de Parataxónomos in Costa Rica (Central America). The suffix -*i* added to the term is a masculine Latin genitive.

**Taxa**. The epithet has been used in two genera: *Alphomelonduvalierbricenoi* Fernández-Triana & Shimbori, 2023; and *Apantelesduvalierbricenoi* Fernández-Triana, 2014.

**Category.** People.

***elegans***

**Etymology.** The Latin word *elegans* means elegant or distinguished. In taxonomy, *elegans* describes a species whose general appearance is elegant, graceful, or refined.

**Taxa.** The epithet has been used in two genera: *Microgasterelegans* Herrich-Schäffer, 1838; and *Microplitiselegans* Timon-David, 1944.

**Category.** Morphology.

**Note.***Microplitiselegans* Timon-David, 1944 is a species only known from fossils (see more detail in Fernández-Triana et al. 2020).

***eliethcantillanoae***

**Etymology.** The epithet refers to Elieth Cantillano for her contributions to the Área de Conservación Guanacaste (ACG) in the Programa de Parataxónomos in Costa Rica (Central America). The suffix -*ae* added to the term is a feminine Latin genitive.

**Taxa**. The epithet has been used in two genera: *Alphomeloneliethcantillanoae* Fernández-Triana & Shimbori, 2023; and *Apanteleseliethcantillanoae* Fernández-Triana, 2014.

**Category.** People.

***epaphus***

**Etymology.** There is no clear explanation of the species name, but it is assumed that corresponds to the butterfly *Siproetaepaphus* Latreille (Nymphalidae), the herbivore host used by these parasitoid wasps.

**Taxa.** The epithet has been used in two genera: *Choerasepaphus* (Nixon, 1965); and *Nyereriaepaphus* (de Saeger, 1944).

**Category.** Biology.

***eros***

**Etymology.** The Greek word *eros* refers to the god of love in Greek mythology. Its Roman counterpart is *Cupido*. In taxonomy, *eros* refers to the lepidopteran host, *Luthrodespandava* Horsfield (Lycaenidae) commonly known as Plain’s cupid.

**Taxa.** The epithet has been used in two genera: *Dolichogenideaeros* (Wilkinson, 1932); and *Parapanteleseros* Gupta, 2014.

**Category.** Biology.

***erro***

**Etymology.** The Latin word *erro* probably means to make a mistake, to err, or go astray. However, there is not a clear meaning or association in the etymology.

**Taxa.** The epithet has been used in two genera: *Diolcogastererro* (Nixon, 1965); and *Microgastererro* Nixon, 1968.

**Category.** Other.

***eupolis***

**Etymology.** The Greek name *eupolis* likely means good city or well-city. The epithet may intend to refer to a specific location or city, but it is difficult to say for certain.

**Taxa.** The epithet has been used in two genera: *Apanteleseupolis* Nixon, 1965; and *Microgastereupolis* Nixon, 1968.

**Category.** Other.

***felipechavarriai***

**Etymology.** The name refers to Felipe Chavarría in recognition of his diligent efforts to understand the plant biology of the Área de Conservación Guanacaste (ACG) in Costa Rica (Central America). The suffix -*i* added to the term is a masculine Latin genitive.

**Taxa.** The epithet has been used in two genera: *Apantelesfelipechavarriai* Fernández-Triana, 2014; and *Snelleniusfelipechavarriai* Fernández-Triana & Whitfield, 2015.

**Category.** People.

***feltiae***

**Etymology.** The term *feltiae* refers to the moth genus, *Feltia* Walker (Noctuidae: Noctuinae) which is used as an herbivore host by these parasitoid wasps. The suffix -*ae* added to the term is a Latin feminine genitive.

**Taxa.** The epithet has been used in two genera: *Apantelesfeltiae* Viereck, 1912; and *Microplitisfeltiae* Muesebeck, 1922.

**Category.** Biology.

***ferruginea***

**Etymology.** The Latin word *ferruginea* derives from *ferrugo* which means rust. In taxonomy, *ferruginea* describes a species that has a reddish brown or rust-colored appearance or a particular morphological trait that resembles rust.

**Taxa.** The epithet has been used in two genera: *Cotesiaferruginea* (Marshall, 1885); and *Microgasterferruginea* Xu & He, 2000.

**Category.** Morphology.

***flavipes***

**Etymology.** The term *flavipes* derives from two Latin words, *flavus* which means yellow and *pes* which means foot. In taxonomy, *flavipes* describes a species with yellow-colored legs. In some cases, the name may refer to the entire organism having a yellow coloration, but it is most commonly used to describe a specific body part.

**Taxa.** The epithet has been used in three genera: *Chaoaflavipes* Luo, You & Xiao, 2004; *Cotesiaflavipes* Cameron, 1891; and *Diolcogasterflavipes* (Haliday, 1834).

**Category.** Morphology.

***garygibsoni***

**Etymology.** The name refers to Gary Alfred Peter Gibson, a Canadian expert in the systematics of chalcid parasitoid wasps (Chalcidoidea), especially the families Eupelmidae and Pteromalidae and functional and comparative morphology of Chalcidoidea and Hymenoptera. The suffix -*i* added to the term is a masculine Latin genitive.

**Taxa.** The epithet has been used in two genera: *Apantelesgarygibsoni* Fernández-Triana, 2014; and *Glyptapantelesgarygibsoni* Arias-Penna, 2019.

**Category.** People.

***ghesquierei***

**Etymology.** The epithet honors Fernand Ghesquière (1879–1924), a Belgian braconidologist and naturalist who made significant contributions to the study of African insects at the end of the 19^th^ and beginning of the 20^th^ century. The suffix -*i* added to the term is a masculine Latin genitive.

**Taxa.** The epithet has been used in two genera: *Apantelesghesquierei* de Saeger, 1941; and *Forniciaghesquierei* de Saeger, 1942.

**Category.** People.

***gloriasihezarae***

**Etymology.** The epithet refers to Gloria Sihezar for her contributions to the Área de Conservación Guanacaste (ACG) in the Programa de Parataxónomos in Costa Rica (Central America). The suffix -*ae* added to the term is a feminine Latin genitive.

**Taxa**. The epithet has been used in two genera: *Alphomelongloriasihezarae* Fernández-Triana & Shimbori, 2023; and *Apantelesgloriasihezarae* Fernández-Triana, 2014.

**Category.** People.

***grangeri***

**Etymology.** The epithet is dedicated to Charles Granger, a French entomologist who started the laborious revision of braconid wasps of the Malagasy subregion. The suffix -*i* added to the term is a masculine Latin genitive.

**Taxa.** The epithet has been used in two genera: *Diolcogastergrangeri* (Shenefelt, 1973); and *Dodogastergrangeri* Rousse, 2013.

**Category.** People.

***guillermopereirai***

**Etymology.** The epithet refers to Guillermo Pereira for his contributions to the Área de Conservación Guanacaste (ACG) in the Programa de Parataxónomos in Costa Rica (Central America). The suffix -*i* added to the term is a feminine Latin genitive.

**Taxa**. The epithet has been used in two genera: *Alphomelonguillermopereirai* Fernández-Triana & Shimbori, 2023; and *Apantelesguillermopereirai* Fernández-Triana, 2014.

**Category.** People.

***hazelcambroneroae***

**Etymology.** The epithet refers to Hazel Cambronero for her contributions to the Área de Conservación Guanacaste (ACG) in the Programa de Parataxónomos in Costa Rica (Central America). The suffix -*ae* added to the term is a feminine Latin genitive.

**Taxa**. The epithet has been used in two genera: *Alphomelonhazelcambroneroae* Fernández-Triana & Shimbori, 2023; and *Apanteleshazelcambroneroae* Fernández-Triana, 2014.

**Category.** People.

***hyphantriae***

**Etymology.** The term *hyphantriae* is derived from the Greek words *hyphantes* which means weaver and *tria* which means three. The suffix -*ae* added to the term is a Latin feminine genitive. In taxonomy, *hyphantriae* refers to a moth genus, *Hyphantria* Harris (Erebidae), which is an herbivore host used by these parasitoid wasps.

**Taxa.** The epithet has been used in two genera: *Cotesiahyphantriae* (Riley, 1887); and *Microplitishyphantriae* Ashmead, 1898.

**Category.** Biology.

***indica***

**Etymology.** The Latin word *indica* derives from *indicus* which means of India. In taxonomy, *indica* refers to a species that is native to or has a significant presence in the Indian subcontinent. The name has been also used to describe morphological traits that are commonly found in organisms from India or the surrounding areas.

**Taxa.** The epithet has been used in three genera: *Apantelesindica* Chougale, 2016; *Cotesiaindica* Sathe & Rokade, 2005; and *Diolcogasterindica* (Wilkinson, 1927).

**Category.** Geography.

**Note.***Apantelesindica* Chougale, 2016 is under the status of unavailable names (see more detail in Fernández-Triana et al. 2020).

***indicus***

**Etymology.** The Latin word *indicus* means of India or belonging to India. In taxonomy, *indicus* refers to a species that is native to or has a significant presence in the Indian subcontinent.

**Taxa.** The epithet has been used in three genera: *Microplitisindicus* Marsh, 1978; *Parapantelesindicus* (Bhatnagar, 1950); and *Pholetesorindicus* Ahmad, Ghramh & Pandey, 2020.

**Category.** Geography.

***insularis***

**Etymology.** The Latin word *insularis* derives from *insula*, which means island or of or belonging to an island. In taxonomy, *insularis* describes a species that is found exclusively or predominantly on islands. The epithet also associates the physical or ecological traits of islands themselves, such as insular climates or insular vegetation.

**Taxa.** The epithet has been used in two genera: *Apantelesinsularis* Muesebeck, 1921; and *Diolcogasterinsularis* (Hedqvist, 1965).

**Category.** Geography.

***isidrochaconi***

**Etymology.** The epithet refers to Isidro Villegas in recognition of his diligent efforts on the Programa de Sectores for the Área de Conservación Guanacaste (ACG) in Costa Rica (Central America). The suffix -*i* added to the term is a masculine Latin genitive.

**Taxa.** The epithet has been used in two genera: *Apantelesisidrochaconi* Fernández-Triana, 2014; and *Snelleniusisidrochaconi* Fernández-Triana & Whitfield, 2015.

**Category.** People.

***jamesi***

**Etymology.** The epithet is dedicated to James Bryan Whitfield, an American braconidologist. He is one of the prestigious authorities on the taxonomy and phylogenomics of microgastrine parasitoid wasps and their coevolution with symbiotic polydnaviruses. The suffix -*i* added to the term is a masculine Latin genitive.

**Taxa.** The epithet has been used in two genera: *Jimwhitfieldiusjamesi* Fernández-Triana & Boudreault, 2018; and *Microplitisjamesi* Austin & Dangerfield, 1993.

**Category.** People.

***jesusugaldei***

**Etymology.** The epithet refers to Jesús Ugalde in recognition of his diligent efforts in the administration of the Instituto Nacional de Biodiversidad (INBio) in Costa Rica (Central America). The suffix -*i* added to the term is a masculine Latin genitive.

**Taxa.** The epithet has been used in two genera: *Apantelesjesusugaldei* Fernández-Triana, 2014; and *Glyptapantelesjesusugaldei* Arias-Penna, 2019.

**Category.** People.

***jorgehernandezi***

**Etymology.** The epithet is dedicated to Jorge Hernández in recognition of his diligent efforts to understand the plant biology of the Área de Conservación Guanacaste (ACG), Costa Rica (Central America). The suffix -*i* added to the term is a masculine Latin genitive.

**Taxa.** The epithet has been used in two genera: *Apantelesjorgehernandezi* Fernández-Triana, 2014; and *Microplitisjorgehernandezi* Fernández-Triana & Whitfield, 2015.

**Category.** People.

***kasparyani***

**Etymology.** The epithet honors the Russian entomologist Dmitri Rafaelievich Kasparyan, for his contributions to ichneumonid taxonomy. The suffix -*i* added to the term is a masculine Latin genitive.

**Taxa.** The epithet has been used in two genera: *Cotesiakasparyani* (Tobias, 1976); and *Diolcogasterkasparyani* Kotenko, 2007.

**Category.** People.

***keineraragoni***

**Etymology.** The epithet refers to Keiner Aragón for his contributions to the Área de Conservación Guanacaste (ACG) in the Programa de Parataxónomos in Costa Rica (Central America). The suffix -*i* added to the term is a feminine Latin genitive.

**Taxa**. The epithet has been used in two genera: *Alphomelonkeineraragoni* Fernández-Triana & Shimbori, 2023; and *Apanteleskeineraragoni* Fernández-Triana, 2014.

**Category.** People.

***keralensis***

**Etymology.** The term *keralensis* consists of two words, Kerala, a state on the Malabar coast of India, and the Latin suffix -*ensis* which means belonging to or inhabiting. Kerala comes from the Malayalam word Keralam which means land of coconuts, the place is known for its rich biodiversity and is home to a large number of native plants and animals. In taxonomy, *keralensis* describes a species that is native to or has a significant presence in Kerala.

**Taxa.** The epithet has been used in two genera: *Illidopskeralensis* (Narendran & Sumodan, 1992); and *Philoplitiskeralensis* Ranjith & Fernández-Triana, 2019.

**Category.** Geography.

***lacteus***

**Etymology.** The Latin word *lacteus* means milky or white as milk. In taxonomy, *lacteus* describes a species that has a milky or white coloration or appearance.

**Taxa.** The epithet has been used in two genera: *Apanteleslacteus* (Nees, 1834); and *Microplitislacteus* Austin & Dangerfield, 1993.

**Category.** Morphology.

***lamprosemae***

**Etymology.** The epithet refers to the moth genus, *Lamprosema* Hübner (Crambidae), which is the herbivore host used by these parasitoid wasps. The suffix -*ae* added to the term is a Latin feminine genitive.

**Taxa.** The epithet has been used in two genera: *Glyptapanteleslamprosemae* (Wilkinson, 1928); and *Illidopslamprosemae* (Ahmad, 2005).

**Category.** Biology.

***longiantenna***

**Etymology.** The term *longiantenna* contains two Latin words, *longus* which means long and *antenna* which means yard, sailyard, or pole. In taxonomy, *longiantenna* describes a species with relatively long antennae compared to other members of the same taxonomic rank.

**Taxa.** The epithet has been used in two genera: *Apanteleslongiantenna* Chen & Song, 2004; and *Fornicialongiantenna* Luo & You, 2008.

**Category.** Morphology.

***longicalcar***

**Etymology.** The term *longicalcar* integrates two Latin words, *longus* which means long and *calcar* which means spur. In taxonomy, *longicalcar* is often used to describe a species with elongated hind leg spurs or tarsal claws which are used for various purposes (e.g., defense, mating, or gripping surfaces).

**Taxa.** The epithet has been used in two genera: *Dolichogenidealongicalcar* (Thomson, 1895); and *Microgasterlongicalcar* Xu & He, 2003.

**Category.** Morphology.

***longivena***

**Etymology.** The term *longivena* contains two Latin words, *longus* which means long and *vena* which means vein. In taxonomy, *longivena* describes a species with elongated veins, structures that help to support the delicate wing during flight.

**Taxa.** The epithet has been used in three genera: *Dolichogenidealongivena* Liu & Chen, 2018; *Glyptapanteleslongivena* Chen & Song, 2004; and *Rasivalvalongivena* Song & Chen, 2004.

**Category.** Morphology.

***loretta***

**Etymology.** The term *loretta* is a female given name of Italian origin. The name derives from the Latin word *laurus* which means laurel. The laurel plant was a symbol of victory, honor, or fame. In taxonomy, *loretta* is used to honor loved ones.

**Taxa.** The epithet has been used in two genera: *Choerasloretta* (Nixon, 1965); and *Distatrixloretta* Grinter, 2009.

**Category.** People.

***lunata***

**Etymology.** The Latin word *lunata* derives from *luna* which means moon, the celestial body that has a crescent shape during certain phases of its cycle. In taxonomy, *lunata* describes morphological traits that have a curved or crescent shape, such as wings or antennae.

**Taxa.** The epithet has been used in three genera: *Apanteleslunata* Song & Chen, 2004; *Cotesialunata* (Packard, 1881); and *Dolichogenidealunata* Liu & Chen, 2019.

**Category.** Morphology.

***malshri***

**Etymology.** The term *malshri* could be a reference to a person’s name based on the suffix -*i*, which is a masculine Latin genitive. It could also refer to a Hindu name for a woman. Malshri in Sanskrit means wonderful garland. The information is incomplete, so it is difficult to determine the exact origin.

**Taxa.** The epithet has been used in two genera: *Cotesiamalshri* (Sathe & Inamdar, 1991); and *Glyptapantelesmalshri* Sathe, Inamdar & Dawale, 2003.

**Category.** People.

**Note.***Glyptapantelesmalshri* Sathe, Inamdar & Dawale, 2003 is under the status of unavailable names (see more detail in Fernández-Triana et al. 2020).

***manuelriosi***

**Etymology.** The epithet refers to Manuel Ríos for his contributions to the Área de Conservación Guanacaste (ACG) in the Programa de Parataxónomos in Costa Rica (Central America). The suffix -*i* added to the term is a masculine Latin genitive.

**Taxa**. The epithet has been used in two genera: *Alphomelon manuel­riosi* Fernández-Triana & Shimbori, 2023; and *Apantelesmanuelriosi* Fernández-Triana, 2014.

**Category.** People.

***masneri***

**Etymology.** The epithet honours Lubomír (Lubo) Masner, a prominent Canadian entomologist, whose work contributes to the understanding of the taxonomy and systematics of the order Hymenoptera. The suffix -*i* added to the term is a masculine Latin genitive.

**Taxa.** The epithet has been used in three genera: *Microplitismasneri* Austin & Dangerfield, 1993; *Philoplitismasneri* Fernández-Triana & Goulet, 2009; and *Pholetesormasneri* (Mason, 1981).

**Category.** People.

***masoni***

**Etymology.** The epithet honors the Canadian William Richardson Miles Mason (1921–1991), one of the greatest pioneers in the study of the Microgastrinae of the 20^th^ century. His legacy is immense. The suffix -*i* added to the term is a masculine Latin genitive.

**Taxa.** The epithet has been used in nine genera: *Apantelesmasoni* Chen & Song, 2004; *Diolcogastermasoni* Saeed, Austin & Dangerfield, 1999; *Dolichogenideamasoni* Pandey, Ahmad, Haider & Shujauddin, 2005; *Hypomicrogastermasoni* Valerio, 2015; *Parapantelesmasoni* Austin & Dangerfield, 1992; *Pholetesormasoni* Whitfield, 2006; *Prasmodonmasoni* Fernández-Triana & Whitfield, 2014; *Sathonmasoni* Williams, 1988; and *Wilkinsonellusmasoni* Long & van Achterberg, 2011.

**Category.** People.

***medon***

**Etymology.** The Greek word *medon* means ruler or lord. In taxonomy, *medon* denotes a dominant, superior, or distinctive morphological trait such as larger size.

**Taxa.** The epithet has been used in two genera: *Apantelesmedon* Nixon, 1965; and *Diolcogastermedon* (Nixon, 1965).

**Category.** Morphology.

***memorata***

**Etymology.** The term *memorata* derives from the Latin word *memoratus* which means remembered or noted. In taxonomy, *memorata* likely emphasizes a notable or remarkable morphological trait present in the species.

**Taxa.** The epithet has been used in two genera: *Iconellamemorata* Kotenko, 2007; and *Microgastermemorata* Papp, 1971.

**Category.** Morphology.

***merata***

**Etymology.** The word Latin adjective *merata* likely means earned or deserved. However, *merate*, *meratus* is also an Australian aboriginal word that means naked. In *Diolcogaster*, *merata* refers to the tergite 3 which does not form a carapace with tergites 1 and 2.

**Taxa.** The epithet has been used in two genera: *Diolcogastermerata* Saeed, Austin & Dangerfield, 1999; and *Iconellamerata* (Kotenko, 1981).

**Category.** Morphology.

***mikepoguei***

**Etymology.** The epithet honors Michael (Mike) G. Pogue, an American lepidopterist. His work has been widely recognized and respected within the lepidopterist community, making his name synonymous with excellence. The suffix -*i* added to the term is a masculine Latin genitive.

**Taxa.** The epithet has been used in two genera: *Glyptapantelesmikepoguei* Arias-Penna, 2019; and *Prasmodonmikepoguei* Fernández-Triana & Whitfield, 2014.

**Category.** People.

***mikesharkeyi***

**Etymology.** The epithet honors the American braconidologist, Michael (Mike) Joseph Sharkey, whose work contributes to the knowledge of Braconidae of the world. He is a distinguished hymenopterologist and his work is truly remarkable. The suffix -*i* added to the term is a masculine Latin genitive.

**Taxa.** The epithet has been used in two genera: *Alphomelonmikesharkeyi* Fernández-Triana & Shimbori, 2023; and *Glyptapantelesmikesharkeyi* Arias-Penna, 2019.

**Category.** People.

***minor***

**Etymology.** The Latin adjective *minor* means smaller or lesser. In taxonomy, *minor* describes a species with a small body size.

**Taxa.** The epithet has been used in two genera: *Apantelesminor* Fahringer, 1938; and *Glyptapantelesminor* Ashmead, 1906.

**Category.** Morphology.

***montanus***

**Etymology.** The Latin adjective *montanus* derives from the noun *mons*, *montis* which means mountain. In taxonomy, *montanus* indicates that the species is of the mountains or has been reported at high altitudes.

**Taxa.** The epithet has been used in two genera: *Apantelesmontanus* de Saeger, 1944; and *Microplitismontanus* Muesebeck, 1922.

**Category.** Geography.

**Note.***Apantelesmontanus* de Saeger, 1944 is under the status of species inquirendae (see more detail in Fernández-Triana et al. 2020).

***narendrani***

**Etymology.** The epithet honors Thekke Curuppathe Narendran (1944–2013), an Indian entomologist, in recognition of his immense contributions to parasitic Hymenoptera. The suffix -*i* added to the term is a masculine Latin genitive.

**Taxa.** The epithet has been used in three genera: *Diolcogasternarendrani* Rema & Sheeba, 2004; *Microplitisnarendrani* Ranjith & Nasser, 2015; and *Neoclarkinellanarendrani* Veena, 2014.

**Category.** People.

***newguineaensis***

**Etymology.** The term *newguineaensis* contains two words, New Guinea, the world’s second-largest island located in the southwestern Pacific Ocean, and the Latin suffix -*ensis* which means belonging to or inhabiting. In taxonomy, *newguineaensis* refers to a species that is native to or was first discovered in New Guinea.

**Taxa.** The epithet has been used in two genera: *Diolcogasternewguineaensis* Saeed, Austin & Dangerfield, 1999; and *Microplitisnewguineaensis* Austin & Dangerfield, 1993.

**Category.** Geography.

***niger***

**Etymology.** The Latin word *niger* means black. In taxonomy, *niger*, *nigra*, *nigrum* are used to describe a species with black or dark coloration on the whole body or in a specific morphological trait.

**Taxa.** The epithet has been used in two genera: *Dolichogenideaniger* (Muesebeck, 1921); and *Jenopappiusniger* (de Saeger, 1944).

**Category.** Morphology.

**Note.** The original species name *Apantelesniger* Muesebeck, 1921, was transferred to *Dolichogenidea* by Fernández-Triana et al. (2020). The specific epithet, *niger*, was changed to *nigra* to comply with gender agreement as the gender of *Dolichogenidea* is feminine (Fernández-Triana et al. 2020).

***nigricornis***

**Etymology.** The term *nigricornis* contains two Latin words, *niger* which translates to black and *cornu* which means horn. In taxonomy, *nigricornis* refers to the black coloration of the antennae or other horn-like structures.

**Taxa.** The epithet has been used in two genera: *Glyptapantelesnigricornis* (Muesebeck, 1921); and *Microgasternigricornis* Motschoulsky, 1863.

**Category.** Morphology.

**Note.***Microgasternigricornis* Motschoulsky, 1863 is under the status of species inquirendae (see more detail in Fernández-Triana et al. 2020).

***nigritus***

**Etymology.** The Latin term *nigritus* means blackened or blackish. In taxonomy, as well as the epithets *niger*, *nigritus* describe a species with black or dark coloration on the whole body or in a specific morphological trait.

**Taxa.** The epithet has been used in two genera: *Hygroplitisnigritus* Luo & You, 2005; and *Microplitisnigritus* Muesebeck, 1922.

**Category.** Morphology.

***nixoni***

**Etymology.** The epithet honors the British entomologist Gilbert Edward James Nixon, one of the most recognized and illustrious experts of Microgastrinae. The suffix -*i* added to the term is a masculine Latin genitive.

**Taxa.** The epithet has been used in five genera: *Apantelesnixoni* Song, 2002; *Diolcogasternixoni* Saeed, Austin & Dangerfield, 1999; *Microgasternixoni* Austin & Dangerfield, 1992; *Prasmodonnixoni* Fernández-Triana & Whitfield, 2014; and *Semionisnixoni* Tobias, 1987.

**Category.** People.

**Note.***Semionisnixoni* Tobias, 1987 is a species only known from fossils (see more detail in Fernández-Triana et al. 2020).

***oculatus***

**Etymology.** The Latin adjective *oculatus* means having eyes or with eyes. In taxonomy, *oculatus* describes a species with distinctive eye markings or coloration around the eyes.

**Taxa.** The epithet has been used in two genera: *Apantelesoculatus* Tobias, 1967; and *Palaeomicrogasteroculatus* Belokobylskij, 2014.

**Category.** Morphology.

**Note.***Palaeomicrogasteroculatus* Belokobylskij, 2014 is a species only known from fossils (see more detail in Fernández-Triana et al. 2020).

***orientalis***

**Etymology.** The term *orientalis* comes from the Latin word *oriens* which means east or rising. In taxonomy, *orientalis* indicates a species that is native to the eastern region of its distribution or was originally described from specimens collected in the East.

**Taxa.** The epithet has been used in six genera: *Apantelesorientalis* Szépligeti, 1913; *Bulukaorientalis* Chou, 1985; *Cotesiaorientalis* Chalikwar & Nikam, 1984; *Diolcogasterorientalis* (Rao & Chalikwar, 1970); *Miropotesorientalis* Fernández-Triana & van Achterberg, 2014; and *Tobleroniusorientalis* Fernández-Triana & Boudreault, 2018.

**Category.** Geography.

***oryzae***

**Etymology.** The term *oryzae* derives from *Oryza* L., (Poaceae), the genus to which the rice belongs. The suffix -*ae* added to the term is a Latin feminine genitive. Many adult microgastrine species have been associated with rice plants or rice grains.

**Taxa.** The epithet has been used in two genera: *Dolichogenideaoryzae* Bhoje & Sathe, 2002; and *Exoryzaoryzae* (Walker, 1994).

**Category.** Other.

**Note.***Dolichogenideaoryzae* Bhoje & Sathe, 2002 is under the status of unavailable name (see more detail in Fernández-Triana et al. 2020).

***osvaldoespinozai***

**Etymology.** The epithet refers to Osvaldo Espinoza for his contributions to the Área de Conservación Guanacaste (ACG) in the Programa de Parataxónomos in Costa Rica (Central America). The suffix -*i* added to the term is a masculine Latin genitive.

**Taxa**. The epithet has been used in two genera: *Alphomelonosvaldoespinozai* Fernández-Triana & Shimbori, 2023; and *Apantelesosvaldoespinozai* Fernández-Triana, 2014.

**Category.** People.

***pappi***

**Etymology.** The name honors the Hungarian entomologist Jenő Papp (1933–2017) in recognition of his significant contributions to the knowledge of Braconidae of the world, and his work on Palearctic Microgastrinae. The suffix -*i* added to the term is a masculine Latin genitive.

**Taxa.** The epithet has been used in two genera: *Choeraspappi* Narendran, 1998; and *Cotesiapappi* Inanç, 2002.

**Category.** People.

**Note.***Choeraspappi* Narendran, 1998 is under the status of species inquirendae (see more detail in Fernández-Triana et al. 2020).

***paranaensis***

**Etymology.** The term *paranaensis* contains two words, Paraná, of the Paraná River in South America, which runs through Brazil, Paraguay, and Argentina, and the Latin suffix -*ensis* which means belonging to or inhabiting. In taxonomy, *paranaensis* refers to a species that is found in or near the Paraná River region.

**Taxa.** The epithet has been used in two genera: *Illidopsparanaensis* Penteado-Dias & Scatolini, 2000; and *Sendaphneparanaensis* Scatolini & Penteado-Dias, 1999.

**Category.** Geography.

***peckorum***

**Etymology.** The term *peckorum* contains two words, Peck honors the married couple Stewart Blaine Peck and Jarmila Kukalová-Peck, tireless insect collectors during the last 30+ years, their contributions are significant to the study of the insect fauna of southern Florida, USA. And the suffix -*orum* which indicates the genitive plural, is typically used to honor a group of people, such as a family or a team of researchers.

**Taxa.** The epithet has been used in two genera: *Keylimepiepeckorum* Fernández-Triana, 2016; and *Papantelespeckorum* Mason, 1981.

**Category.** People.

***peruensis***

**Etymology.** The term *peruensis* contains two words, Peru, a country in South America, and the Latin suffix -*ensis* which means belonging to or inhabiting. In taxonomy, *peruensis* indicates that the species is from Peru.

**Taxa.** The epithet has been used in two genera: *Snelleniusperuensis* Shenefelt, 1968; and *Venanusperuensis* Mason, 1981.

**Category.** Geography.

***petronariosae***

**Etymology.** The epithet refers to Petrona Ríos for her contributions to the Área de Conservación Guanacaste (ACG) in the Programa de Parataxónomos in Costa Rica (Central America). The suffix -*ae* added to the term is a feminine Latin genitive.

**Taxa**. The epithet has been used in two genera: *Alphomelon petronari­osae* Fernández-Triana & Shimbori, 2023; and *Apantelespetronariosae* Fernández-Triana, 2014.

**Category.** People.

***phildevriesi***

**Etymology.** The epithet honors Philip (Phil) James DeVries, an American tropical ecologist, for his diligent contributions to this field. The suffix -*i* added to the term is a masculine Latin genitive.

**Taxa.** The epithet has been used in two genera: *Glyptapantelesphildevriesi* Arias-Penna, 2019; and *Snelleniusphildevriesi* Fernández-Triana & Whitfield, 2015.

**Category.** People.

***philippinensis***

**Etymology.** The term *philippinensis* contains two words, the Philippine, an archipelagic country in southeast Asia, and the Latin suffix -*ensis* which means belonging to or inhabiting. In taxonomy, *philippinensis* refers to a species that is native or discovered in the Philippines.

**Taxa.** The epithet has been used in two genera: *Glyptapantelesphilippinensis* (Ashmead, 1904); and *Snelleniusphilippinensis* (Ashmead, 1904).

**Category.** Geography.

***phthorimaeae***

**Etymology.** The term *phthorimaeae* contains two words, *phthorima* a Greek word that means destruction or ruin, and the Latin suffix -*ae*, a feminine genitive. In taxonomy, *Phthorimaea* Meyrick refers to a gelechiid moth genus that is used as an herbivore host by these wasps and that may cause potential damage to their food plants.

**Taxa.** The epithet has been used in two genera: *Dolichogenideaphthorimaeae* (Muesebeck, 1921); and *Microgasterphthorimaeae* Muesebeck, 1922.

**Category.** Biology.

***pinicola***

**Etymology.** The term *pinicola* contains two Latin words, *pinus* which means pine and *cola* which means inhabitant of or dweller. In taxonomy, *pinicola* refers to a species that is commonly associated with pine trees or dwelling in pines.

**Taxa.** The epithet has been used in two genera: *Glyptapantelespinicola* (Lyle, 1917); and *Venanuspinicola* Mason, 1981.

**Category.** Biology.

***prodeniae***

**Etymology.** The term *prodeniae* contains two words, *Prodenia* Guenée, a noctuid moth genus, (currently, is a synonym of *Spodoptera* Guenée), and the Latin suffix -*ae*, a feminine genitive.

**Taxa.** The epithet has been used in two genera: *Dolichogenideaprodeniae* (Viereck, 1912); and *Microplitisprodeniae* Rao & Kurian, 1950.

**Category.** Biology.

***punctata***

**Etymology.** The Latin word *punctata* means spotted or dotted. In taxonomy, *punctata* refers to a species that has visible dots or spots or a specific type of surface sculpturing (punctate) on its body.

**Taxa.** The epithet has been used in two genera: *Diolcogasterpunctata* (Rao & Chalikwar, 1976); and *Neoclarkinellapunctata* Ahmad, Pandey, Haider & Shujauddin, 2005.

**Category.** Morphology.

***pyrene***

**Etymology.** The epithet probably refers to the butterfly species, *Ixiaspyrene* Linnaeus (Pieridae) which is used as the herbivore host by these wasps. However, it is necessary to confirm the etymology.

**Taxa.** The epithet has been used in two genera: *Diolcogasterpyrene* (Nixon, 1965); and *Iconellapyrene* (Nixon, 1965).

**Category.** Biology.

***randallgarciai***

**Etymology.** The epithet is dedicated to Randall García in recognition of his key role in the founding of the Área de Conservación Guanacaste (ACG) and subsequent diligent efforts in the administration of the Instituto Nacional de Biodiversidad (INBio) in Costa Rica (Central America). The suffix -*i* added to the term is a masculine Latin genitive.

**Taxa.** The epithet has been used in two genera: *Apantelesrandallgarciai* Fernández-Triana, 2014; and *Venanusrandallgarciai* Fernández-Triana & Whitfield, 2014.

**Category.** People.

***rarus***

**Etymology.** The Latin adjective *rarus* means rare or uncommon. In taxonomy, *rarus* indicates a species that is rare or has a limited distribution.

**Taxa.** The epithet has been used in two genera: *Parapantelesrarus* Valerio & Whitfield, 2009; and *Semionisrarus* Nixon, 1965.

**Category.** Other.

***ricardocaleroi***

**Etymology.** The epithet refers to Ricardo Calero for his contributions to the Área de Conservación Guanacaste (ACG) in the Programa de Parataxónomos in Costa Rica (Central America). The suffix -*i* added to the term is a masculine Latin genitive.

**Taxa**. The epithet has been used in two genera: *Alphomelonricardocaleroi* Fernández-Triana & Shimbori, 2023; and *Apantelesricardocaleroi* Fernández-Triana, 2014.

**Category.** People.

***robertoespinozai***

**Etymology.** The epithet is dedicated to Roberto Espinoza in recognition of his diligent efforts for the Programa de Parataxónomos and the plant inventory of the Área de Conservación Guanacaste (ACG), Costa Rica (Central America). The suffix -*i* added to the term is a masculine Latin genitive.

**Taxa.** The epithet has been used in two genera: *Apantelesrobertoespinozai* Fernández-Triana, 2014; and *Snelleniusrobertoespinozai* Fernández-Triana & Whitfield, 2015.

**Category.** People.

***rodrigogamezi***

**Etymology.** The epithet honors Rodrigo Gámez in recognition of his enormous efforts in support of founding the Área de Conservación Guanacaste (ACG), and founding and directing the Instituto Nacional de Biodiversity (INBio) in Costa Rica (Central America). The suffix -*i* added to the term is a masculine Latin genitive.

**Taxa.** The epithet has been used in two genera: *Apantelesrodrigogamezi* Fernández-Triana, 2014; and *Pseudapantelesrodrigogamezi* Fernández-Triana & Whitfield, 2014.

**Category.** People.

***rogerblancoi***

**Etymology.** The epithet is dedicated to Roger Blanco in recognition of his diligent efforts for the Programa de Investigacion and administration of the Área de Conservación Guanacaste (ACG) in Costa Rica (Central America). The suffix -*i* added to the term is a masculine Latin genitive.

**Taxa.** The epithet has been used in three genera: *Apantelesrogerblancoi* Fernández-Triana, 2014; *Dolichogenidearogerblancoi* Fernández-Triana & Boudreault, 2019; and *Sendaphnerogerblancoi* Fernández-Triana & Whitfield, 2014.

**Category.** People.

***ronaldzunigai***

**Etymology.** The epithet refers to Ronald Zúñiga in recognition of his diligent efforts for the Programa de Parataxónomos of the Área de Conservación Guanacaste (ACG) and Hymenoptera curatorial taxonomy for Instituto Nacional de Biodiversidad (INBio) in Costa Rica (Central America). The suffix -*i* added to the term is a masculine Latin genitive.

**Taxa.** The epithet has been used in two genera: *Apantelesronaldzunigai* Fernández-Triana, 2014; and *Glyptapantelesronaldzunigai* Arias-Penna, 2019.

**Category.** People.

***rostermoragai***

**Etymology.** The epithet refers to Roster Moraga for his contributions to the Área de Conservación Guanacaste (ACG) in the Programa de Parataxónomos in Costa Rica (Central America). The suffix -*i* added to the term is a masculine Latin genitive.

**Taxa**. The epithet has been used in two genera: *Alphomelonrostermoragai* Fernández-Triana & Shimbori, 2023; and *Apantelesrostermoragai* Fernández-Triana, 2014.

**Category.** People.

***ruficoxis***

**Etymology.** The term *ruficoxis* contains two Latin words, *rufus* which means red or reddish and *coxa* which means hip or thigh. In taxonomy, *ruficoxis* refers to the reddish coloration of the coxa.

**Taxa.** The epithet has been used in two genera: *Cotesiaruficoxis* (Hedwig, 1962); and *Microgasterruficoxis* Ruthe, 1858.

**Category.** Morphology.

**Note.***Microgasterruficoxis* Ruthe, 1858 is under the status of species inquirendae (see more detail in Fernández-Triana et al. 2020).

***rufipes***

**Etymology.** The term *rufipes* contains two Latin words, *rufus* which means red and *pes* which means foot. In taxonomy, *rufipes* describe a species with red-colored legs.

**Taxa.** The epithet has been used in two genera: *Microgasterrufipes* Nees, 1834; and *Microplitisrufipes* Dutu-Lacatusu, 1961.

**Category.** Morphology.

***rufithorax***

**Etymology.** The term *rufithorax* contains two Latin words, *rufus* which means red or reddish, and *thorax* which means chest. In taxonomy, *rufithorax* refers to a species with a reddish-coloured metasoma or thorax.

**Taxa.** The epithet has been used in two genera: *Apantelesrufithorax* Hedqvist, 1965; and *Diolcogasterrufithorax* (Granger, 1949).

**Category.** Morphology.

***rufiventris***

**Etymology.** The term *rufiventris* derives from two Latin words, *rufus* which means reddish and *venter* which means abdomen, belly, or stomach. In taxonomy, *rufiventris* is commonly used to describe a species with a reddish-colored abdomen, metasoma, or ventral side.

**Taxa.** The epithet has been used in two genera: *Cotesiarufiventris* (Bingham, 1906); and *Microplitisrufiventris* Kokujev, 1914.

**Category.** Morphology.

***rugosa***

**Etymology.** The Latin word *rugosa* means wrinkled or rough. In taxonomy, *rugosa* describes a species that exhibits a specific type of surface sculpturing (rugose) on the body or body part, meaning a wrinkled appearance.

**Taxa.** The epithet has been used in three genera: *Cotesiarugosa* (Szépligeti, 1914); *Hypomicrogasterrugosa* Valerio, 2015; and *Rasivalvarugosa* (Muesebeck, 1922).

**Category.** Morphology.

***rugulosus***

**Etymology.** The term *rugulosus* is a Latin adjective that means wrinkled or rugose and derives from the noun *ruga* which means a wrinkle. In taxonomy, *rugulosus* describes a species with a body or a body part showing a specific type of surface sculpturing (rugulose). In other words, an appearance of roughness or wrinkledness.

**Taxa.** The epithet has been used in two genera: *Choerasrugulosus* Song & Chen, 2014; and *Hygroplitisrugulosus* (Nees, 1834).

**Category.** Morphology.

***schizurae***

**Etymology.** The term *schizurae* refers to a notodontid moth genus, *Schizura* Doubleday (currently a synonym of *Coelodasys* Packard). The suffix -*ae* added to the term is a Latin feminine genitive. In taxonomy, *schizurae* refers to the herbivore host used by these wasps.

**Taxa.** The epithet has been used in two genera: *Cotesiaschizurae* (Ashmead, 1898); and *Diolcogasterschizurae* (Muesebeck, 1922).

**Category.** Biology.

***scottmilleri***

**Etymology.** The epithet honors Scott Everett Miller, an American entomologist for his pioneering research in the systematics of Lepidoptera (moths); biogeography of the Pacific Basin, New Guinea, and Africa; and plant-insect community ecology. The suffix -*i* added to the term is a masculine Latin genitive.

**Taxa.** The epithet has been used in two genera: *Glyptapantelesscottmilleri* Arias-Penna, 2019; and *Prasmodonscottmilleri* Fernández-Triana & Whitfield, 2014.

**Category.** People.

***sergioriosi***

**Etymology.** The epithet refers to Sergio Ríos for his contributions to the Área de Conservación Guanacaste (ACG) in the Programa de Parataxónomos in Costa Rica (Central America). The suffix -*i* added to the term is a masculine Latin genitive.

**Taxa**. The epithet has been used in two genera: *Alphomelonsergioriosi* Fernández-Triana & Shimbori, 2023; and *Apantelessergioriosi* Fernández-Triana, 2014.

**Category.** People.

***seyrigi***

**Etymology.** The epithet refers to André Seyrig, a French entomologist for his groundbreaking work on the taxonomy of Hymenoptera. The suffix -*i* added to the term is a masculine Latin genitive.

**Taxa.** The epithet has been used in three genera: *Apantelesseyrigi* Wilkinson, 1936; *Diolcogasterseyrigi* (Granger, 1949); and *Forniciaseyrigi* Granger, 1949.

**Category.** People.

***shivranginii***

**Etymology.** The term *shivranginii* possibly refers to Shivaranjani, a Hindustani classical raga (melodic mode) in Indian classical music. The Latin suffix -*i* added also suggests that the term refers to a male person. Another possibility is that the epithet has some local or personal significance. Unfortunately, no information was available in the original description or subsequent literature.

**Taxa.** The epithet has been used in two genera: *Parapantelesshivranginii* Sathe & Ingawale, 1989; and *Protomicroplitisshivrangini* Sathe, Inamdar & Dawale, 2003.

**Category.** People.

**Note.***Protomicroplitisshivranginii* Sathe, Inamdar & Dawale, 2003 is under the status of unavailable names (see more detail in Fernández-Triana et al. 2020).

***shrii***

**Etymology.** The term *shrii* refers to the Sanskrit word *Shri* which is often used as a prefix to convey respect or reverence, equivalent to the English Mr. or Ms. It is often used as an honorific title for deities, holy persons, and respected leaders.

**Taxa.** The epithet has been used in two genera: *Apantelesshrii* Sathe & Ingawale, 1995; and *Cotesiashrii* Sathe, Ingawale & Bhosale, 1994.

**Category.** Other.

**Note.***Apantelesshrii* Sathe & Ingawale, 1995 is under the status of species inquirendae (see more detail in Fernández-Triana et al. 2020).

***siderion***

**Etymology.** The term *siderion* comes from the Greek word *sideros* which means iron. In taxonomy, *siderion* describes a species that is associated with iron-rich environments or exhibits metallic coloration resembling iron.

**Taxa.** The epithet has been used in two genera: *Glyptapantelessiderion* (Nixon, 1965); and *Hypomicrogastersiderion* Valerio, 2015.

**Category.** Morphology.

***similis***

**Etymology.** The Latin word *similis* means similar or like. In taxonomy, *similis* indicates a species that is closely related to another in appearance or behavior.

**Taxa.** The epithet has been used in two genera: *Microplitissimilis* Lyle, 1921; and *Snelleniussimilis* Long & van Achterberg, 2013.

**Category.** Other.

***spodopterae***

**Etymology.** The epithet *spodopterae* refers to a moth genus, *Spodoptera* Guenée (Noctuidae), which is used as the herbivore host by these parasitoid wasps. In turn, *Spodoptera* derives from the Greek word *spodos* meaning ash wood, and the Latin suffix -*ptera*, which means winged. The suffix -*ae* added to the term is a Latin feminine genitive.

**Taxa.** The epithet has been used in two genera: *Glyptapantelesspodopterae* Ahmad, 2009; and *Microplitisspodopterae* Rao & Kurian, 1950.

**Category.** Biology.

***striatus***

**Etymology.** The Latin term *striatus* derives from the verb *strio* which means to mark with lines. In taxonomy, *striatus* describes a species with stripes or lines on their bodies or appendages.

**Taxa.** The epithet has been used in three genera: *Keylimepiestriatus* (Muesebeck, 1922); *Philoplitisstriatus* Fernández-Triana & Goulet, 2009; and *Wilkinsonellusstriatus* Austin & Dangerfield, 1992.

**Category.** Morphology.

***subcamilla***

**Etymology.** The term *subcamilla* contains two Latin words, the prefix -*sub* which means below or under, and *Camilla*, a character that appears in the Aeneid, an epic poem written by Virgil.

**Taxa.** The epithet has been used in two genera: *Apantelessubcamilla* Long, 2007; and *Iconellasubcamilla* (Tobias, 1976).

**Category.** Other.

***sunflowari***

**Etymology.** The term *sunflowari* contains two words, sunflower, the common name of the genus *Helianthus* L. (Asteraceae), and the suffix -*i*, an adjectival ending used to form species names. In taxonomy, *sunflowari* refers to the food plant of these parasitoid wasps.

**Taxa.** The epithet has been used in two genera: *Cotesiasunflowari* Sathe & Bhoje, 2000; and *Dolichogenideasunflowari* Sathe & Bhoje, 2000.

**Category.** Other.

**Note.** Both species are under the status of unavailable names (see more detail in Fernández-Triana et al. 2020).

***szelenyii***

**Etymology.** The word refers to Gusztáv Szelényi (1904–1982), a Hungarian zoologist and entomologist from the 20^th^ century for his outstanding contributions. The suffix -*i* added to the term is a masculine Latin genitive.

**Taxa.** The epithet has been used in two genera: *Dolichogenideaszelenyii* (Papp, 1972); and *Microgasterszelenyii* Papp, 1974.

**Category.** People.

***taiwanensis***

**Etymology.** The term *taiwanensis* contains two parts, Taiwan (officially the Republic of China), a country in East Asia, and the Latin suffix -*ensis* which means belonging to or of the place of. In taxonomy, *taiwanensis* refers to a species that was first identified or discovered in Taiwan or is endemic to Taiwan.

**Taxa.** The epithet has been used in three genera: *Bulukataiwanensis* Austin, 1989; *Dolichogenideataiwanensis* (Sonan, 1942); and *Pholetesortaiwanensis* Liu & Chen, 2016.

**Category.** Geography.

***taylori***

**Etymology.** The term *taylori* probably refers to Taylor, a surname of English and Scottish origin, which is one of the most common surnames in English-speaking countries. The suffix -*i* added to the term is a masculine Latin genitive.

**Taxa.** The epithet has been used in two genera: *Glyptapantelestaylori* (Wilkinson, 1928); and *Microplitistaylori* Austin & Dangerfield, 1993.

**Category.** People.

***testacea***

**Etymology.** The term *testaceous* comes from the Latin word *testa* which means a piece of burned clay, a shell, or a potsherd. In taxonomy, *testaceous* refers to the dull, brick-red color of fired clay or terracotta, and later came to refer to any of the several pale colors of bricks. Over time, the term has been used to describe a range of colors, from pale pink to pale brown, which resemble the color of fired clay.

**Taxa.** The epithet has been used in two genera: *Cotesiatestacea* Fujie, Shimizu & Fernández-Triana, 2018; and *Dolichogenideatestacea* Liu & Chen, 2018.

**Category.** Morphology.

***thoseae***

**Etymology.** The term *thoseae* refers to a moth genus, *Thosea* Walker (Limacodidae), which is used as an herbivore host by these parasitoid wasps. The suffix -*ae* added to the term is a Latin feminine genitive. The genus name *Thosea* presumably derives from the Greek word *thos*, which means a run, a course, or a career, possibly about the way the caterpillar moves like slugs or snails.

**Taxa.** The epithet has been used in two genera: *Forniciathoseae* Wilkinson, 1930; and *Glyptapantelesthoseae* (Wilkinson, 1934).

**Category.** Biology.

***tobiasi***

**Etymology.** The epithet honors Vladimir Ivanovich Tobias (1929–2011), a Russian hymenopterologist, for his pioneering work on the systematics of Braconidae. The suffix -*i* added to the term is a masculine Latin genitive.

**Taxa.** The epithet has been used in three genera: *Dolichogenideatobiasi* (Balevski, 1980); *Microplitistobiasi* Kotenko, 2007; and *Wilkinsonellustobiasi* Long, 2007.

**Category.** People.

***townesi***

**Etymology.** The epithet is dedicated to Henry Keith Townes, an American entomologist, for his significant contributions to our understanding of the family Ichneumonidae. The suffix -*i* added to the term is a masculine Latin genitive.

**Taxa.** The epithet has been used in two genera: *Apantelestownesi* Nixon, 1965; and *Bulukatownesi* Austin, 1989.

**Category.** People.

***typhon***

**Etymology.** The Greek word *typhon* refers to *Typhon* (also *Typhoeus*, *Typhaon*, *Thyphos*), a giant serpentine monstrous and the deadliest creature in Greek mythology known as the Father of All Monsters. *Typhon* probably reflects the fiery nature and the destructive power of these parasitoids attacking the pests.

**Taxa.** The epithet has been used in two genera: *Alloplitistyphon* Nixon, 1965; and *Promicrogastertyphon* (Nixon, 1965)

**Category.** Other.

***urios***

**Etymology.** There is no specific information available on the origin of the term *urios*. Presumably, the term *urios* has the epithet *apertus* which means open, obvious, or uncover. Another possibility is that the word *urios* comes from the Messapic language, an extinct Indo-European Paleo-Balkanic language of the southeastern Italian Peninsula, that means doors.

**Taxa.** The epithet has been used in two genera: *Diolcogasterurios* (Nixon, 1965); and *Glyptapantelesurios* Kotenko, 2007.

**Category.** Other.

**Note.***Glyptapantelesurios* Kotenko, 2007 is under the status of *Nomina nuda* (see more detail in Fernández-Triana et al. 2020). Other.

***variabilis***

**Etymology.** The term *variabilis* derives from the Latin word *varius* which means varying or diverse. In taxonomy, *variabilis* describes a species that exhibits significant variation in its morphological traits.

**Taxa.** The epithet has been used in two genera: *Kiwigastervariabilis* Fernández-Triana & Ward, 2011; and *Pholetesorvariabilis* Whitfield, 2006.

**Category.** Morphology.

***varicolor***

**Etymology.** The term *varicolor* contains two Latin words, *varius* which means diverse or varied and *color* which means color. In taxonomy, *varicolor* describes a species with variable or changing colors.

**Taxa.** The epithet has been used in two genera: *Choerasvaricolor* Song & Chen, 2014; and *Microplitisvaricolor* Viereck, 1917.

**Category.** Morphology.

***vietnamensis***

**Etymology.** The term *vietnamensis* contains two words, Vietnam, a country in Southeast Asia, and the Latin suffix -*ensis* which means belonging to or inhabiting. In taxonomy, *vietnamensis* indicates a species that is native to Vietnam.

**Taxa.** The epithet has been used in two genera: *Paroplitisvietnamensis* van Achterberg & Fernández-Triana, 2013; and *Ungunicusvietnamensis* Fernández-Triana & Boudreault, 2018.

**Category.** Geography.

***vitobiasi***

**Etymology.** The epithet is dedicated to Vladimir Ivanovich Tobias (the first two letters (vi) refer to his two names), a Russian hymenopterologist, for his pioneering work on the systematics of Braconidae. The suffix -*i* added to the term is a masculine Latin genitive.

**Taxa.** The epithet has been used in two genera: *Illidopsvitobiasi* Kotenko, 2004; and *Microplitisvitobiasi* Fernández-Triana, 2019.

**Category.** People.

***wittei***

**Etymology.** The epithet honors Gaston François de Witte, a Belgian herpetologist, who led the expedition to Africa during which the wasps-type series were collected. The suffix -*i* added to the term is a masculine Latin genitive.

**Taxa.** The epithet has been used in two genera: *Diolcogasterwittei* (de Saeger, 1944); and *Dolichogenideawittei* (de Saeger, 1944).

**Category.** People.

***yanayacuensis***

**Etymology.** The term *yanayacuensis* contains two words, Yanayacu, a Biological Station and Center for Creative Studies, an area with 100 hectares in the cloud forest of Ecuador (South America), and the Latin suffix -*ensis* which means belonging to or of the place of.

**Taxa.** The epithet has been used in three genera: *Alphomelonyanayacuensis* Fernández-Triana & Shimbori, 2023; *Glyptapantelesyanayacuensis* Arias-Penna, 2019; and *Venanusyanayacuensis* Arias-Penna & Whitfield, 2011.

**Category.** Geography.

***yeimycedenoae***

**Etymology.** The epithet is dedicated to Yeimy Cedeño Solis in recognition of her management of the Refugio Nacional de Vida Silvestre Ostional in the Área de Conservación Tempisqué (ACT), Costa Rica (Central America).

**Taxa.** The epithet has been used in two genera: *Dolichogenideayeimycedenoae* Fernández-Triana & Boudreault, 2019; and *Exoryzayeimycedenoae* Fernández-Triana, 2016.

**Category.** People.

***yunnanensis***

**Etymology.** The term *yunnanensis* contains two words, Yunnan, a landlocked province in the southwest of China, and the Latin suffix -*ensis* which means belonging to or of the place of. In taxonomy, *yunnaensis* refers to a species native to Yunnan.

**Taxa.** The epithet has been used in three genera: *Choerasyunnanensis* Song & Chen, 2014; *Microgasteryunnanensis* Xu & He, 1999; and *Protapantelesyunnanensis* (You & Xiong, 1987).

**Category.** Geography.

***zhaoi***

**Etymology.** The term *zhaoi* refers to Zhao, the most traditional surname in the Chinese language. The suffix -*i* added to the term is a masculine Latin genitive. In taxonomy, *zhaoi* possibly honors a male Chinese entomologist.

**Taxa.** The epithet has been used in two genera: *Microgasterzhaoi* Xu & He, 1997; and *Microplitiszhaoi* Xu & He, 2000.

**Category.** People.

## ﻿Discussion

**Figure 4.**
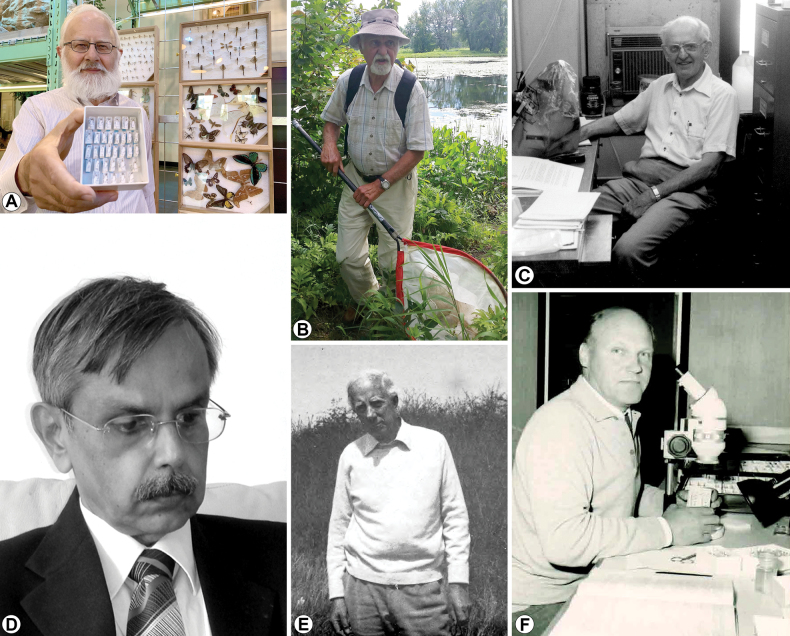
Photographs of the prominent entomologist, whose names have been chosen multiple times to name species of Microgastrinae (Hymenoptera, Braconidae) **A** Cornelis (Kees) van Achterberg (Courtesy of Naturalis Biodiversity Center, Leiden, Netherlands) **B** Lubomír Masner (Credit: Carolyn Trietsch) **C** William Richardson Miles Mason (Courtesy of Canadian National Collection of Insects, Arachnids and Nematodes (CNC) of Agriculture and Agri-Food Canada, Ottawa) **D** Thekke Curuppathe Narendran (Courtesy of Indian Academy of Sciences, Bengaluru) **E** Gilbert Edward James Nixon (based on an edited photo from Huddleston 1988) **F** Vladimir Ivanovich Tobias (based on an edited photo from Belokobylskij and Sharkey 2011).

Within the subfamily Microgastrinae, the level of repetition of specific epithets was low. This indicates that there is a high degree of originality by the descriptors at the time of naming a new species. In other words, the authors have designated an original specific epithet for each new species they describe. Reviewing the taxonomic history, the approaches in species naming within the subfamily follow the traditional taxonomy practices, most epithets refer mainly to external morphological traits (morphology) and species distribution (geography). Nevertheless, the use of people’s names has significantly increased in the 20^th^ century (e.g., Guedes et al. 2023), although historically, a substantial portion of specific epithets was also attributed to people. These names primarily provide a way to pay homage to collectors and scientists. These are the cases of the Canadian William RM Mason and the English Gilbert EJ Nixon. They are among the most prominent entomologists whose contributions laid the foundation of knowledge of the subfamily Microgastrinae (Fig. 4); Mason in the Nearctic Region and Nixon in the Palearctic Region. Their influence has endured to the present day and this explains why the specific epithets *masoni* and *nixoni* are among the most repeated.

In recent years, the use of etymologies to pay tribute to artists, celebrities, sportsmen, politicians, or even loved ones has been striking in several taxonomic groups. However, within Microgastrinae, etymologies are mainly addressed to people who have had direct involvement in recent long-term projects. Those projects form an intricate network encompassing technicians (e.g., collectors, database managers, parataxonomists) and specialists (e.g., botanist, ecologist, environmentalist, geneticist, hymenopterologist, lepidopterologist) whose collaboration has been immortalized in the names of the described species (e.g., *Pseudapanteleslaurachinchillae* Fernández-Triana & Whitfield, in Fernández-Triana et al. 2014). Some of those collaborative collecting efforts have been developed in Central America (in northwestern Costa Rica), South America (Colombia, the Eastern Andes in Ecuador), Asia (Iran, Philippines), and Pacific Ocean islands (Fiji) among others.

The presence of the largest specific epithets in the morphology, people, and geography categories is partly explained by the use of compound words. In these three categories, most of the specific epithets are created by linking two words. Thus, in morphology, the use of a noun (referring to the structure) + the adjective is frequent; to honor people, the use of the name + the surname is added most of the time, and for geography, a noun (place) + the suffix -*ensis* is used regularly.

It is well known that the Achilles’ heel in the subfamily Microgastrinae is the lack of a sound phylogeny. However, another vulnerability is the deficiency of natural history knowledge (e.g., both lepidopteran hosts and food plants). The insufficiency of biological information could partly explain why the biology category is the one with the lowest number of species with repeated specific epithets (26 species out of 303).

It is important to acknowledge that these aforementioned conclusions are confined to the scope of identical specific epithets and the outcomes derived from the present study, and thus do not encompass the entirety of species belonging to this subfamily.

## ﻿Recommendations

The taxonomist’s creativity in conceiving a specific epithet is inspirational, and it prompts us to reflect on how scientific knowledge is communicated. Taxonomic confusion increases when a specific epithet is repeated. For that reason, it is important to reduce it as much as possible. In essence, the recurrence of specific epithets that are identical and initially described in different genera does not pose a particular issue and the International Code of Zoological Nomenclature accommodates the admissibility of numerous instances where species names are identical, as per its provisions. The sole concern arises when a species is transferred to another genus that already bears the same specific name, resulting in the emergence of two homonymous names (Articles 57–60). Such instances lead to an increase in the number of homonyms and the subsequent proliferation of new names within the taxa. It should be noted that the concept homonym established by the ICZN is followed here and not other alternative terms such as asthenomonym (secondary homonym), and hadromonym (primary homonym), known as subcategories of homonym, proposed by Linz Zoological Committee (LZC: https://skosmos.loterre.fr/th63/en/) (Dubois 2011).

**Figure 5.**
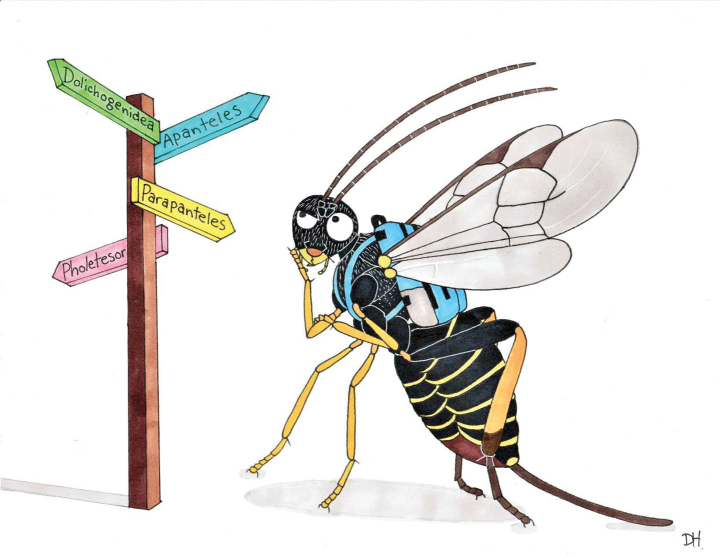
A microgastrine parasitoid wasp, with a potential identity crisis (e.g., the specific epithet, *masoni*), carrying a backpack, is observed studying a signpost with arrows pointing to different close genera. The parasitoid wasp has a befuddled expression as she contemplates which path to take. The Canadian artist, Devon Henderson, sketched the cartoon in response to our request (Waspaholic Comics).

As shown in Fig. 5, there is one potential scenario for consideration of transferring *Pholetesormasoni* to *Parapanteles* based on the existing evidence (Fernández-Triana et al. 2020). Should this nomenclatural change take place, it would lead to homonymy, necessitating the replacement of the epithet *masoni* with a new name. As a precautionary measure, it is advisable to refrain from assigning a species name that duplicates one already present in a closely related genus, considering the potential future scenario of the two genera being recognized as synonymous and/or homonyms. The prevalence of identical specific epithets within certain taxonomic groups presents a modest inconvenience as it may lead to potential confusion among taxonomists. Nonetheless, from our perspective, this issue is relatively minor in Microgastrinae and can be effectively managed without significant ramifications. Hence, the matter of species name registration within a unified database (e.g., TaxonWorks: https://taxonworks.org) offers a promising opportunity for taxonomists. Simple actions, such as verifying the provisional specific epithet across all available information (consolidated database and/or original descriptions) can prevent epithet replication and ensure accurate representation of the species under description. While the overall occurrence of duplicated specific epithets in the entire subfamily is minimal, it is advised to persist in avoiding duplicate names and minimizing identical epithets. To ensure the utmost clarity in nomenclature, researchers must endeavor to choose unique and distinctive specific epithets.

Here are some recommendations to enhance the accuracy of species datasets and minimize redundances:

- Taxonomic authorities should consider adopting a standardized nomenclature for species names to prevent the use of the same specific epithets for species within the same group.
- Researchers should verify and reconcile data sources to ensure accuracy, and timeliness, and to prevent overlapping or conflicting records.
- Data management systems should utilize quality control measures to detect and eliminate duplicate names, such as employing automated algorithms or conducting manual inspections.
- Collaborative data sharing between researchers, institutions, and organizations can aid in detecting and resolving species dataset duplication issues.
- Further research is necessary to comprehend the causes of duplication in species name datasets. This can include analyzing the data sources, scrutinizing the taxonomic literature, and exploring the ecological and biogeographical effects of duplication.

By implementing these recommendations, taxonomists could improve the accuracy of species name datasets while reducing duplication and improving the proper understanding of species diversity, distribution, and ecology. Adopting these recommended practices would lead to improved data quality and promote more reliable taxonomic research and analysis. Reducing duplication and enhancing understanding of species attributes would improve clarity and precision in scientific investigations. This would lead to better-informed conservation strategies, ecological assessments, and biodiversity management efforts. Implementing these recommendations would ultimately advance the field of taxonomy, enabling a more robust and accurate representation of the natural world.

## ﻿Additional information

### Author ORCIDs

Mostafa Ghafouri Moghaddam https://orcid.org/0000-0002-1942-9689

Diana Carolina Arias-Penna https://orcid.org/0000-0002-9601-3038

Minoo Heidari Latibari https://orcid.org/0000-0003-4158-8034

Buntika A. Butcher https://orcid.org/0000-0002-0541-0709

### Data availability

All of the data that support the findings of this study are available in the main text.
